# The Use of MRI-Derived Radiomic Models in Prostate Cancer Risk Stratification: A Critical Review of Contemporary Literature

**DOI:** 10.3390/diagnostics13061128

**Published:** 2023-03-16

**Authors:** Linda My Huynh, Yeagyeong Hwang, Olivia Taylor, Michael J. Baine

**Affiliations:** 1 Department of Radiation Oncology, Fred & Pamela Buffett Cancer Center, University of Nebraska Medical Center, 987521 Nebraska Medical Center, Omaha, NE 68198-7521, USA; 2 Department of Urology, University of California, Orange, CA 92868, USA

**Keywords:** prostate cancer, radiomics, risk stratification

## Abstract

The development of precise medical imaging has facilitated the establishment of radiomics, a computer-based method of quantitatively analyzing subvisual imaging characteristics. The present review summarizes the current literature on the use of diagnostic magnetic resonance imaging (MRI)-derived radiomics in prostate cancer (PCa) risk stratification. A stepwise literature search of publications from 2017 to 2022 was performed. Of 218 articles on MRI-derived prostate radiomics, 33 (15.1%) generated models for PCa risk stratification. Prediction of Gleason score (GS), adverse pathology, postsurgical recurrence, and postradiation failure were the primary endpoints in 15 (45.5%), 11 (33.3%), 4 (12.1%), and 3 (9.1%) studies. In predicting GS and adverse pathology, radiomic models differentiated well, with receiver operator characteristic area under the curve (ROC-AUC) values of 0.50–0.92 and 0.60–0.92, respectively. For studies predicting post-treatment recurrence or failure, ROC-AUC for radiomic models ranged from 0.73 to 0.99 in postsurgical and radiation cohorts. Finally, of the 33 studies, 7 (21.2%) included external validation. Overall, most investigations showed good to excellent prediction of GS and adverse pathology with MRI-derived radiomic features. Direct prediction of treatment outcomes, however, is an ongoing investigation. As these studies mature and reach potential for clinical integration, concerted effort to validate these radiomic models must be undertaken.

## 1. Introduction

In recent years, the advancement of precise medical imaging has facilitated the establishment of radiomics, a computer-based method of extracting and quantitatively analyzing subvisual imaging characteristics [1]. Radiomic features (i.e., qualities of intensity, texture, shape, or wavelet) can be extracted from a variety of medical images (computed tomography (CT), magnetic resonance imaging (MRI), or positron emission tomography (PET) images) using advanced mathematical algorithms, aggregated into predictive models, and applied to enhance personalized therapy [2,3]. The radiomics pipeline includes (1) image acquisition and preprocessing, (2) high-throughput feature extraction, and (3) data integration and data analysis. This process ultimately results in a predictive or prognostic model based on extracted radiomic features and can be applied to any clinical endpoint. Although the methodology behind radiomics is rapidly evolving, several contemporary studies have highlighted the enormous potential of radiomics in a variety of diseases, including, but not limited to, cancers of the gastrointestinal tract [4,5], lung [6], brain [7], and genitourinary tract [8,9]. 

In prostate cancer (PCa) diagnosis, preoperative MRI is standard of care and most commonly used for treatment planning and prediction of adverse pathology [10,11]. In this regard, the literature to date highlights the use of MRI-derived radiomics as an extension of this purpose. Radiomic models are most often reported to be utilized in the prediction of high-risk pathology, high Gleason score (GS), and, more recently, treatment failure following surgery or radiation [1]. As PCa is highly heterogeneous, the identification of imaging-based biomarkers predictive of clinical outcomes would enable disease-tailored treatment planning and prediction of therapy response independent of tissue biopsy and molecular analysis. If applied to identify men at high risk for recurrence, for example, these models could enhance discussion on treatment strategy, tolerable risks and benefits to the patient, and the need or lack thereof for individual therapies. In this regard, the aim of the present review is to summarize the current literature on the use of diagnostic MRI-derived radiomics in PCa risk stratification via prediction of GS, adverse pathology, and postsurgical recurrence or postradiation failure. 

## 2. Methods

A stepwise literature search of publications from 2017 to 2022 was performed. A search of Medical Literature Analysis and Retrieval System Online (MEDLINE) databases was completed utilizing the following keywords and combination(s) thereof: [radiomics] with/without [prostate cancer] or [prostate], interchanged with [mpMRI] and/or [MRI]. This yielded 218 articles. A hand-search was performed for articles assessing the use of diagnostic MRIs (defined as scans obtained prior to any treatment) in PCa risk stratification (defined with endpoints of Gleason score (GS), postsurgical high-risk pathology (i.e., extraprostatic extension (EPE), positive surgical margin (PSM), and/or lymph node invasion (LNI)), postsurgical recurrence, and/or postradiation therapy failure). If a study included other endpoints outside of the aforementioned, the study was still included in this review but data from other endpoints were not reported. Non-English publications, review articles, editorials, and commentaries were excluded, but the reference list of each was searched to ensure inclusion of all relevant studies. 

Utilizing the following stepwise methodology, studies were reviewed by the study team for inclusion and exclusion criteria defined a priori. First, the titles and abstracts were screened such that nonrelevant studies were excluded. Second, full manuscripts were reviewed for their study populations and/or outcome measures. Two authors (LH and MB) independently agreed on the selection of eligible studies and achieved consensus of included studies. Data on the number of subjects, outcome measures, image series used, feature selection, region of interest, and model validation were systematically extracted from each article and summarized in Table 1, Table 2, Table 3 and Table 4. To ensure standardization, the International Society of Urological Pathology (ISUP) guidelines on GS [12], National Comprehensive Center Network (NCCN) and American Urological Association (AUA) guidelines for risk group stratification [11,13], and Prostate Imaging Reporting and Data System version 2 (PI-RADS v2) were utilized [14]. 

## 3. Results

### 3.1. Study Selection

Two-hundred and eighteen publications were initially identified and screened through a literature search of the MEDLINE journals via a PubMed interface. Of these, 52 review articles, 1 letter to the editor, and 1 clinical trial protocol were excluded, leaving 164 records for title and abstract review. After title and abstract review, 112 records were excluded for clinical endpoints outside of risk stratification for Gleason grade, adverse pathology, and post-treatment recurrence or failure, leaving 52 for full-paper review. Nineteen additional records were excluded during full-paper review, as they did not utilize diagnostic MRIs at the time of PCa diagnosis (*n* = 4), predicted biopsy Gleason grade (*n* = 7), predicted presence of overt radiographic features for increased staging (*n* = 5), or utilized registries without clinical endpoints (*n* = 3). After all inclusion and exclusion criteria were satisfied, 33 articles remained and were reviewed. 

### 3.2. Description of Studies

Of the 33 included articles, all studies were published between 2017 and 2022, with the majority (*n* = 26, 78.7%) published in 2020 or later. Overall, in image acquisition and preprocessing, T2 weighted images (T2WI) were universally used, with diffusion-weighted images (DWI) and apparent diffusion coefficient (ADC) sequences in the second majority. Prediction of GS and adverse pathology (i.e., PSM, EPE, and/or LNI) postradical prostatectomy (RP) were primary endpoints in 15 (45.5%) and 11 (33.3%) studies, respectively. Four (12.1%) and three (9.1%) investigations highlighted the use of MRI-derived radiomics in predicting postsurgical recurrence and postradiation failure, respectively. Finally, of the 33 studies included, 7 (21.2%) reported external validation of their radiomic models. 

#### 3.2.1. Prediction of Gleason Grade

While many studies have been conducted to utilize MRI-derived radiomic models to discriminate between clinically significant (GS ≥ 3 + 3) [11,13] and insignificant (GS < 3 + 3) PCa lesions, these studies are out of the scope of the current review. Rather, we focused on stratification of clinically significant PCa by GS risk groups [15,16,17]. Table 1 summarizes the 15 studies utilizing MRI-derived radiomic models to predict pathologic GS [15,16,17,18,19,20,21,22,23,24,25,26,27,28,29], with one study predicting GS upgrading between biopsy and surgery [23]. 

To this effect, several of these studies compared the utility of radiomic features extracted from different imaging sequences (T2WI, DWI, ADC, etc.) [21,22,25,29]. Gong et al. found DWI to outperform T2WI image sequences in GS risk stratification, while two other groups combined radiomic features from T2WI and ADC image sequences into their final model [19,21,25]. 

In delineating regions of interest, eight studies [15,17,18,19,22,24,25,27] utilized only PI-RADS lesions, while the remaining seven utilized the full prostate [16,20,21,23,26,28,29]. Feature extraction and selection was most commonly done via minimum redundancy maximum relevance (mRMR) [21,28,29] or random forest (RF) [15,16,24], and the receiver operator characteristic–area under the curve (ROC-AUC) of the final models ranged from 0.50 to 0.92. Castillo and colleagues [27] were the only group to perform a multi-institutional external validation of their model for the prediction of GS ≤ 6 versus GS ≥ 7, yielding an ROC-AUC of 0.75. 

#### 3.2.2. Prediction of Adverse Pathology

Table 2 summarizes the 11 studies utilizing MRI-derived radiomic models to predict EPE, PSM, or LNI [25,30,31,32,33,34,35,36,37,38,39]. Seven [25,31,32,33,38,39], six [25,34,35,36,37,39], and one [38] study(ies) reported on EPE, LNI, and PSM, respectively. Uniquely, Damascelli et al. utilized both EPE and LNI [25] as their primary endpoint, while He and colleagues utilized both PSM and EPE [38]. Furthermore, Li et al. constructed a radiomic model to predict “adverse pathology”, inclusive of any EPE, SVI, and/or LNI [39]. All studies predicting adverse pathology utilized final pathology following RP as the gold standard comparator. 

Of the studies utilizing radiomics to predict EPE, the prostate capsule [30], PI-RADS lesions [31,38], the index lesion [25,33], and prostate [39] were delineated as regions of interest. The ROC-AUC on training sets ranged from 0.674 to 0.92, while the ROC-AUC on testing sets ranged from 0.598 to 0.92. For studies predicting LNI, the prostate [34,37,39], PI-RADS lesions [35,37], and index lesions [25,36] were used as the regions of interest. The ROC-AUC on training sets ranged from 0.82 to 1.0, and the ROC-AUC for testing sets ranged from 0.73 to 0.94. Of note, out of the 11 studies predicting adverse pathology, external validation was pursued in three (27.3%) [32,33,34], of which yielded highly heterogeneous results with ROC-AUC values between 0.598 and 0.80.

In predicting adverse pathology, two studies integrated clinical characteristics with radiomic features to construct a combined model [35,38]. First, a study by Bourbonne and colleagues generated a combined clinical and radiomic model to predict LNI [35]. This model included the six most important radiomic features combined with clinical parameters of tumor size, t-stage, Gleason score, pre- and postoperative prostate-specific antigen (PSA), margin status, age at surgery, and the University of California San Francisco Cancer of the Prostate Risk Assessment Score (UCSF-CAPRA) score. Training of this integrated model yielded an ROC-AUC of 1.0, and model testing in their internal cohort resulted in an ROC-AUC of 0.87. Similarly, a study by He et al. integrated clinical parameters with radiomic models predicting ECE and PSM. Inclusion of these variables increased the ROC-AUC for ECE prediction from 0.625 to 0.728 and increased the ROC-AUC for PSM prediction from 0.733 to 0.766 [38]. Neither of these studies provided correlational analysis between the selected radiomic features and clinical characteristics. 

#### 3.2.3. Prediction of Postsurgical Biochemical Recurrence

To date, there have been only a handful of investigations utilizing radiomic features to predict biochemical recurrence (BCR) following RP. Table 3 illustrates the four studies [39,40,41,42] utilizing preoperative MRI-derived radiomics to predict post-RP BCR, defined as two consecutive serum PSA levels greater than or equal to 0.2 ng/mL. 

Bourbonne et al. was the first to train and validate an MRI-derived radiomic model against post-RP BCR and BCR-free survival [40]. After feature extraction from T2WI and ADC maps of 107 patients, independent factors correlating with BCR were identified via Cox regression analysis. The final radiomic model had a high negative predictive value of 96% and could be reliably used to identify patients at a very low risk of BCR. 

Similar results for BCR prediction at various timepoints were obtained by three other studies, which all included external cohorts in the construction of their radiomic models [39,41,42]. Furthermore, two of these three studies included head-to-head comparisons between their radiomic model and commonly used clinical nomograms for prediction of BCR. Yan et al. reported ROC-AUCs ranging from 0.84 to 0.88 in predicting three-year BCR [41], while Shiradkar et al. reported their radiomic model [42] to be significantly and independently correlated with three-year BCR in cox proportional hazards regression modeling (HR: 2.91, 95% CI: 1.45–11.51, *p* = 0.02). In addition, both studies compared their radiomic models to the UCSF-CAPRA and CAPRA-S scores. Compared to these clinical nomograms, the radiomic model proposed by Yan et al. [41] maintained significantly improved concordance with three-year BCR (*p* < 0.05). While ROC-AUCs for these clinical models ranged from 0.535 to 0.689 across the internal and external datasets, their radiomic signature yielded ROC-AUCs ranging from 0.685 to 0.877. A statistical comparison on this improvement was not reported. These results were similarly echoed by Li and colleagues in 2021 [39], who reported on a combined radiomic-clinicopathologic model (RadClip) integrating radiomic features and clinical characteristics utilized in the UCSF-CAPRA score. In multivariate analysis, RadClip was independently associated with BCR (HR: 7.01, 95% CI: +1.21–40.68, *p* < 0.05). Diagnostic performance via ROC-AUC, sensitivity, and specificity were not reported. 

#### 3.2.4. Prediction of Postradiation Biochemical Failure

Finally, Table 4 summarizes three studies [43,44,45] utilizing pretreatment MRI-derived radiomic models to predict postradiation therapy biochemical failure (BF), defined as a PSA nadir > 2.0 ng/mL. Each of the three studies defined the region of interest differently: one study included the lesion, prostate, peripheral zone, and transitional zone [43], another utilized the prostate and 2 mm of margin [44], and the last contoured only the prostate [45]. In similar regard, each of the three studies predicting postradiation BF approached feature selection and extraction differently. Gnep and colleagues selected five radiomic features most significantly correlated with BF: tumor volume, T2W difference variance mean, T2W contrast mean, maximal tumor area, and ADC median [43]. Combined, these features yielded a radiomic model with a C-index of 0.90 ± 0.09. In contrast, Fernandes et al. developed a radiomic model with T2WI images, predicting BF with an ROC-AUC of 0.63 [44]. Finally, Zhong and colleagues generated a radiomic model with an ROC-AUC of 0.99 in training and an ROC-AUC of 0.73 in validation [45]. Of note, none of these studies included external validation, integration of clinical characteristics with the radiomic models, or head-to-head comparisons with clinical nomograms. 

## 4. Discussion

With the rise of big data, the use of radiomics in personalized medicine is anticipated. More specifically, PCa radiomics is a continuously evolving research field with high potential to offer noninvasive, personalized biomarkers for risk stratification. Expectedly, the number of research articles on MRI-derived prostate radiomics has exponentially increased since 2017, accounting for 218 original articles in the last five years. In our qualitative review of these articles, however, it is clear that only a minority concentrate on the use of MRI-derived radiomics in PCa risk stratification. Rather, most of these investigations have concentrated on its use as a screening or diagnostic tool, for instance, in the correlation of radiomic models with PI-RADS lesions [46], in confirming prostate biopsy findings [47], or as a PCa screening tool [48]. While these are valuable explorations, there is little room for clinical integration of radiomic models in these spaces, as robust gold standards for risk stratification are already present via pathologic examination or imaging. Furthermore, the exploration of radiomic-based models has thus far been independently sequestered within the fields of radiation oncology, radiology, and biomedical imaging. As the use of radiomics in PCa further evolves, multidisciplinary collaborations are necessary to place an indication to the technology. 

A clear focus of PCa radiomics is GS discrimination, reflecting the need for improvements in risk stratification during an initial PCa diagnosis. In this regard, the Prostate Imaging Reporting and Data System (PI-RADS) was validated to identify clinically significant versus insignificant prostate cancer, with prediction rates of up to 82% [49]. However, while PI-RADS works well for the definition of benign versus malignant tissue, it does not differentiate between low-risk and high-risk PCa. Rather, this risk stratification is defaulted to the use of GS on direct prostate biopsy or surgical pathology. 

While GS is the most established histologic biomarker, due to sampling methods and the high intratumoral heterogeneity associated with PCa, GS can be discordant between biopsy and final surgical pathology in 20% to 60% of patients [45,46]. Even further, the risk of upgrading between biopsy and surgery ranges from 5% to 65% in some patient populations [50]. To address this gap, radiomics-based GS prediction may serve as a surrogate measure of tumor heterogeneity and provide an opportunity for further risk stratification between biopsy and initial treatment via surgery or radiation. In this regard, several basic science studies have explored correlations between radiomic features and genetic characteristics of PCa. While these radiogenomic models have not been externally validated, McCann et al. [51] and Switlyk et al. [52] have demonstrated associations between radiomic features and the genetic marker phosphatase and tensin homolog. Similarly, a pilot study by Sun and colleagues correlated radiomic features with hypoxic gene expression—a gene signature identified as an independent risk factor for metastasis-free survival in patients with PCa [53]. With further confirmation correlating radiomic features and genetic drivers of PCa, tumor heterogeneity can be further addressed to facilitate treatment planning. 

Parallel to explorations of GS discrimination are those predicting adverse pathology such as PSM, EPE, and LNI. While preoperative imaging may allow for identification of EPE and LNI, diagnostic performance varies widely, with studies reporting sensitivity and specificity as low as 47% to 56% [54,55,56]. Given that EPE and LNI are associated with significantly decreased likelihood of recurrence-free and progression-free survival [13], early stratification may facilitate conversations regarding multimodal therapy or prompt changes in patient management. 

Finally, as is apparent in the distribution of articles included in this review, direct prediction of treatment outcomes via radiomic models is an investigation still in its infancy. Given the long natural life history unique to PCa, a lengthy follow-up time is required for studies predicting post-RP BCR or postradiation BF. Furthermore, while seven studies have developed models to predict BCR and BF, external validation was only pursued in three, with none utilized in postradiation populations. Prior to clinical integration, further validation efforts are required. A brief review of currently ongoing clinical trials reveals several trials utilizing MRI-derived radiomic signatures for the prediction of discontinuation of active surveillance [56,57], local control rates [58], and disease extension [59]. Furthermore, as explorations of combined imaging techniques utilizing prostate-specific membrane antigen (PSMA) PET/CT continue to evolve, risk stratification may extend further beyond that of the prostate MRI. 

Overall, while most of the included studies in this review presented good-to-excellent ROC-AUC values in predicting GS, adverse pathology, and cancer control, these findings must be considered within the context of publication bias and variability of methodology. First, the development of a radiomic signature consists of several key steps surrounding image acquisition and preprocessing, feature extraction, data integration, and data analysis—each step of which can be modified in statistical methodology, segmentation of regions of interest, cross-validation, testing and validation, and reporting. Given that radiomic features and their corresponding models are highly sensitive to any modifications to these steps, investigations on radiomic feature variability, robustness of available datasets, and reproducibility in multiple cohorts are required prior to consideration of external validation [60,61]. Along similar lines, it is also clear that current studies on PCa radiomics lack comparison to currently clinically available prediction tools or clinical characteristics such as PSA at diagnosis, age, or PI-RADS score. While a few studies have compared their full radiomic models to the CAPRA and CAPRA-S score [39,42], for example, this is a feature in a small minority (<5%) of studies [62]. Even for those investigations integrating clinical characteristics with their radiomic models, there is no analysis to assess possible correlations between each radiomic feature versus already-available clinical information. Until there are adequate comparators between radiomics and information easily obtained during the PCa clinical care pathway, clinical applicability of this technology will be severely hampered. 

## 5. Conclusions

Overall, MRI-derived PCa radiomics presents as an emerging research field with the potential to offer noninvasive, imaging-based biomarkers useful for risk stratification and prediction of treatment response. Furthermore, radiomics has the potential to facilitate quantitative characterization of tumor heterogeneity, thus enabling disease-tailored treatment planning. While radiomic models show promise in predicting high-risk GS and adverse pathology, direct application to prediction of treatment outcomes remains an ongoing investigation. As these studies mature and reach potential for clinical integration, concerted efforts to establish adequate comparators, standardize methodology, and systematically validate these models must be prioritized. 

## Figures and Tables

**Table 1:** Summary of studies utilizing magnetic resonance imaging (MRI)-derived radiomic models to predict Gleason score.

Author	Year	*n*=	Outcome Measure	Images Used	Feature Selection	Region of Interest	Number of Features	Model Validation	Results
Chaddad [15]	2018	99	GS ≤ 6, 3 + 4, ≥4 + 3	T2WI ADC	Random forest (RF)	Lesion	41	4:1 training:testing 5-fold cross-validation	Gleason score ≤ 6, AUC = 0.834 Gleason score = 3 + 4, AUC = 0.727 Gleason score ≥ 4 + 3, AUC = 0.774
Penzias [16]	2018	36	GS 6 vs. >6	T2WI	Random forest (RF)	Prostate Lesion	2001	3-fold cross-validation 23:13 training:testing	Gabor texture features model, AUC = 0.69 Gland lumen shape feature model, AUC = 0.75
Toivonen [17]	2019	100	GS 3 + 3 vs. ≥3 + 3	T2WI DWII	Logistic regression with L1 or L2 regularization	Lesion	ADC 1281/7105 T2WI 1631/7105	Inner 10-fold cross-validation; Outer leave-pair-out cross-validation (LPOCV)	AUC = 0.88
Hectors [18]	2019	64	GS ≥ 8	T2WI ADC	Least absolute shrinkage and selection operator (LASSO)	Lesion	226	Nested cross-validation	Gleason score ≥ 8, AUC = 0.72 Decipher score ≥ 0.6, AUC = 0.84
Abdollahi [19]	2019	33	IMRT response GS ≤ 7 or >7	T2WI ADC	Radiomics module of 3D Slicer SelectKBest_chi2 Variance threshold Select from Model Select Percentile	Lesion	IMRT response: 40/1135 GS score: NR	10-fold cross-validation	Pre-IMRT ADC, AUC = 0.77 for IMRT response T2WI, AUC = 0.0739 for GS prediction ADC, AUC = 0.675 for GS prediction
McGarry [20]	2019	48	GS < 4, GS 4–5	T2WI ADC DCE	Custom algorithm programmed in MATLAB	Prostate	NR	3:1 training:testing 3-fold cross-validation	AUC = 0.79
Gong [21]	2020	489	GS > 7	T2WI DWI	Pyradiomics v3.6 in Python Minimum redundancy maximum relevance (mRMR)	Prostate	Prostate: 1345	Single-sequence	DWI, AUC = 0.801 in training DWI, AUC = 0.787 in testing T2WI, AUC = 0.712 in training T2WI, AUC = 0.645 in testing
Zhang [22]	2020	166	GS upgrading	T2WI ADC DCE	Mutual information maximation (MIM) criterion	Lesion	20/4404	116:50 training:testing	Combined T2WI, ADC, DCE training, AUC = 0.899 Combined T2WI, ADC, DCE testing, AUC = 0.868
Santone [23]	2021	112	GGG 1–5	T2WI	Language of Temporal Ordering Specification (LOTOS)	Area of suspicion	NR	NR	Sensitivity, range 0.95–1.0 Specificity, 1.0
Makowski [24]	2021	85	GS 6, 7, and ≥8	T2WI ADC	R, package tidyverse Random forest (RF) Stochastic gradient boosting (SGB) Support vector machine (SVM) K-nearest neighbor (KNN)	Lesion Peripheral/Transitional zones	322	Cross-validation	SVM model, AUC = 0.92 RF model, AUC = 0.83 SGB model, AUC = 0.75 KNN model, AUC = 0.50
Damascelli [25]	2021	62	GS 7 (3 + 4), 7 (4 + 3), 8, 9	T2WI DWI DCE ADC	Radiomics module of 3D Slicer v4.10.2	Lesion	T2WI features: 44/93 ADC features: 61/93	10-fold cross-validation Training set only	Combined T2WI & ADC model for GGG, AUC = 0.88 Combined T2WI & ADC model for LNI, AUC = 0.90 Combined T2WI & ADC model for EPE, AUC = 0.85
Gugliandolo [26]	2021	49	GS 3 + 4, GS 4 + 3	T2WI	Hierarchical clustering procedure	Prostate	Prostate: 1058/1702	Leave-out cross-validation	AUC = 0.80 in training AUC = 0.75 in testing
Castillo [27]	2021	204	GS ≤ 6, ≥7	T2WI DWI ADC	Workflow for optimal radiomics classification (WORC)	Lesion	540	4:1 training:testing 100× random-split cross-validation	Training: AUC = 0.72, sensitivity = 0.76, specificity = 0.55 Testing: AUC = 0.75, sensitivity = 0.88, specificity = 0.63 External validation: AUC = 0.75
Rodrigues [28]	2021	281	GS > 7	T2WI DWI ADC	Support vector machine weighing (SVM-RFE) Boruta algorithm Minimum redundancy maximum relevance (mRMR) LASSO regularization	Lesion Prostate	Lesion, 167/321 Prostate: 257/321	288 pipelines produced, producing Gland and Lesion datasets	SVM-RFE pipeline, F2 = 0.7226, Kappa = 0.3781 mRMR pipeline, F2 = 0.7071, Kappa = 0.4095 Whole gland, F2 = 0.7426, Kappa = 0.351 Lesion: F2 = 0.6682, Kappa = 0.3687
Gong [29]	2022	489	GS > 7, =7, <7	T2WI DWI ADC	Pyradiomics v3.6 in Python Minimum redundancy maximum relevance (mRMR)	Prostate	3D: 1409/4227 2D: 1046/3138	2:1 training:testing	3D, AUC = 0.800, specificity = 0.781, sensitivity = 0.703 2D, AUC = 0.776, specificity = 0.798, sensitivity = 0.674

**Table 2:** Summary of studies utilizing MRI-derived radiomic models to predict adverse pathology (i.e., positive surgical margins, extraprostatic extension, and lymph node invasion).

Author	Year	*n*=	Outcome Measure	Images Used	Feature Selection	Region of Interest	Number of Features	Model Validation	Results
Ma [30]	2020	119	EPE	T2WI	Least absolute shrinkage and selection operator (LASSO)	Prostate + 1 mm margin	156/1619	10-fold cross-validation 2:1 training:testing 74 training 45 validation	Training: AUC = 0.906, sensitivity = 0.946; specificity = 0.770 Validation: AUC = 0.821, sensitivity = 0.846, specificity = 0.703
Xu [31]	2020	115	EPE	T2WI DWI DCE ADC	Least absolute shrinkage and selection operator (LASSO) Minimum redundancy maximum relevance (mRMR)	Lesions	30	82 training 33 testing	Training AUC = 0.919 Testing AUC = 0.865 Validation AUC = 0.857, sensitivity = 0.714, specificity = 0.895
Bai [32]	2021	284	EPE	T2WI ADC	Least absolute shrinkage and selection operator (LASSO) Minimum redundancy maximum relevance (mRMR)	Peritumoral Intratumoral	19/1595 19/1595	158 internal training 68 internal testing 58 external validation	Peritumoral model AUC = 0.674 Intratumoral model AUC = 0.554 Final model on internal testing AUC = 0.803 Final model on external validation AUC = 0.598
Cuocolo [33]	2021	193	EPE	T2WI DWI ADC	Scikit-learn Python3 package Weka data mining software Synthetic Minority Oversampling Technique	Index lesion	14/2436	10-fold cross-validation 104 training set 43 external test set 46 external validation	Training set AUC = 0.83 External test set AUC = 0.80 External validation set 2 AUC = 0.73
Hou [34]	2021	401	LNI	T2WI DWI ADC	Pyradiomics v3.6 in Python Random forest (RF)	Prostate	NR/2553	280 training 71 internal testing 50 external tests	Training, AUC = 0.93 Internal testing, AUC = 0.92 External testing, AUC = 0.76
Bourbonne [35]	2021	280	LNI	ADC T2WI	Multilayer Perceptron Network, SPSS v24.0	Lesions	6/8640	6:4 training:testing	Combined clinical and radiomic model training AUC = 1.0 Combined clinical and radiomic model testing AUC = 0.87
Zheng [36]	2022	244	LNI	T2WI ADC	PyRadiomics v3.0.1 in Python 3.6 Sequential Floating Forwarding Selection	Index lesion	11	5-fold cross validation 160 training 84 testing	AUC = 0.915; sensitivity = 0.786; specificity = 0.90; NPV = 0.955; PPV = 0.611
Liu [37]	2022	602	LNI	T2WI DWI ADC DCE	ANOVA Recursive feature elimination	Prostate Lesions	19/755 (model 1); 11/829 (model 2); 17/646 (model 3); 16/650 (model 4)	332 training 142 testing	Training cohort: Model 1 (AUC = 0.85, sensitivity = 0.74, specificity = 0.86) Model 2 (AUC = 0.86, sensitivity = 0.71, specificity = 0.91) Model 3 (AUC = 0.94, sensitivity = 0.89, specificity = 0.93) Model 4 (AUC = 0.82, sensitivity = 0.67, specificity = 0.81) Testing cohort: Model 1 (AUC = 0.63, sensitivity = 0.55, specificity = 0.76) Model 2 (AUC = 0.70, sensitivity = 0.69, specificity = 0.67) Model 3 (AUC = 0.73, sensitivity = 0.81, specificity = 0.62) Model 4 (AUC = 0.56, sensitivity = 0.93, specificity = 0.29)
Damascelli [25]	2021	62	LNI EPE	T2WI DWI DCE ADC	Radiomics module of 3D Slicer v4.10.2 Support vector machine model	Index lesion	T2WI features: 44/93 ADC features: 61/93	10-fold cross-validation Training set only	Combined T2WI and ADC model for LNI, AUC = 0.90 Combined T2WI and ADC model for EPE, AUC = 0.85
He [38]	2021	459	PSM EPE	T2WI DWI ADC	Minimum redundancy maximum relevance (mRMR) Least absolute shrinkage and selection operator (LASSO)	Lesions	278 (T2WI) 293 (ADC) EPE prediction 268 (T2WI) 295 (ADC) PSM prediction	7:3 training:testing	ECE, ADC AUC = 0.625 ECE, ADC integrated clinical model AUC = 0.728 PSM, T2WI AUC = 0.614 PSM, ADC AUC = 0.733 PSM, T2WI integrated clinical model AUC = 0.680 PSM, ADC Integrated clinical model AUC = 0.766
Li [39]	2021	198	Adverse pathology, including EPE, SVI, and LNI	T2WI DWI ADC	Minimum redundancy maximum relevance (mRMR)	Prostate	5	5-fold, 10-run cross-validation 71 training 127 testing	Adverse pathology AUC = 0.71

**Table 3:** Summary of studies utilizing MRI-derived radiomic models to predict biochemical recurrence following radical prostatectomy.

Author	Year	*n*=	Outcome Measure	Images Used	Feature Selection	Region of Interest	Number of Features	Model Validation	Results
Bourbonne [40]	2020	195	BCR	T2WI ADC	Multilayer Perceptron Network, SPSS v24.0	Lesion	NR	107 training 88 external testing	Clinical AUC = 0.68 Radiomic AUC = 0.82 Clinical and radiomic AUC = 0.86
Li [39]	2021	198	BCR	T2WI DWI ADC	Minimum redundancy maximum relevance (mRMR)	Prostate	5	5-fold, 10-run cross-validation 71 training 127 testing	Training model HR = 7.01, 95% CI: 1.21–40.68, *p* < 0.05, independent of preoperative and clinicopathologic parameters in multivariable analysis Testing model HR = 1.9, 95% CI: 1.4–2.7, *p* < 0.05
Yan [41]	2021	485	BCR	T2WI	Deep survival radiomic neural network Dense box: three hidden layers with 48 neurons Auto-coding: two hidden layers with 48 neurons + a hidden layer with 24 neurons	Lesions	155/702	368 training 34 external testing 83 external validation	3-year BCR training AUC = 0.84 3-year BCR external testing AUC = 0.85 3-year BCR external validation AUC = 0.84 5-year BCR training AUC = 0.83 5-year BCR external testing AUC = 0.88 5-year BCR external validation AUC = 0.88 Significantly improved accuracy compared to GG-RP, CAPRA-S, NCCN, and CAPRA clinical models
Shiradkar [42]	2022	133	BCR	T2WI ADC	Random forest (RF) Cox proportional hazards regression model	Prostate Lesions	10	Cross-validation 2:1 training:testing	3-year BCR HR: 2.91, 95% CI: 1.45–11.51, *p* = 0.02 Significantly improved accuracy compared to clinical characteristics, CAPRA, CAPRA-S, and Decipher scores

**Table 4:** Summary of studies utilizing MRI-derived radiomic models to predict biochemical failure following radiation therapy.

Author	Year	*n*=	Outcome Measure	Images Used	Feature Selection	Region of Interest	Number of Features	Model Validation	Results
Gnep [43]	2017	74	BF	T2WI ADC	Random forest (RF)	Lesion Prostate Peripheral zone Transitional zone	144	Training only	Five radiomic features C-index 0.90 ± 0.09 (tumor volume, T2W difference variance mean, T2W contrast mean, maximal tumor area, ADC median)
Fernandes [44]	2018	120	BF	T2WI	Minimum redundancy maximum relevance (mRMR) Random forest (RF)	Prostate + 2 mm margin	254	10-fold cross-validation	AUC = 0.63
Zhong [45]	2020	91	BF	T1WI T2WI DWI	Inception-Resnet-v2 network	Prostate	45/1536	3-fold cross-validation 4:1 training:testing	Mean training AUC = 0.99 Mean testing AUC = 0.73

## Data Availability

Not applicable.
